# Liver Glucokinase_**A456V**_ Induces Potent Hypoglycemia without Dyslipidemia through a Paradoxical Induction of the Catalytic Subunit of Glucose-6-Phosphatase

**DOI:** 10.1155/2011/707928

**Published:** 2011-12-13

**Authors:** Anna Vidal-Alabró, Alícia G. Gómez-Valadés, Andrés Méndez-Lucas, Jordi Llorens, Ramon Bartrons, Jordi Bermúdez, Jose C. Perales

**Affiliations:** Biophysics Unit, Department of Physiological Sciences II, IDIBELL-University of Barcelona, Campus de Bellvitge, 08907 L'Hospitalet de Llobregat, Spain

## Abstract

Recent reports point out the importance of the complex GK-GKRP in controlling glucose and lipid homeostasis. Several GK mutations affect GKRP binding, resulting in permanent activation of the enzyme. We hypothesize that hepatic overexpression of a mutated form of GK, GK_A456V_, described in a patient with persistent hyperinsulinemic hypoglycemia of infancy (PHHI) and could provide a model to study the consequences of GK-GKRP deregulation *in vivo*. GK_A456V_ was overexpressed in the liver of streptozotocin diabetic mice. Metabolite profiling in serum and liver extracts, together with changes in key components of glucose and lipid homeostasis, were analyzed and compared to GK wild-type transfected livers. Cell compartmentalization of the mutant but not the wild-type GK was clearly affected *in vivo,* demonstrating impaired GKRP regulation. GK_A456V_ overexpression markedly reduced blood glucose in the absence of dyslipidemia, in contrast to wild-type GK-overexpressing mice. Evidence in glucose utilization did not correlate with increased glycogen nor lactate levels in the liver. PEPCK mRNA was not affected, whereas the mRNA for the catalytic subunit of glucose-6-phosphatase was upregulated ~4 folds in the liver of GK_A456V_-treated animals, suggesting that glucose cycling was stimulated. Our results provide new insights into the complex GK regulatory network and validate liver-specific GK activation as a strategy for diabetes therapy.

## 1. Introduction

Glucokinase (GK) (EC 2.7.1.1) is expressed in glucosensitive cells of the pancreas, liver, hypothalamus, anterior pituitary gland, and enteroendocrine K and L cells [1–3]. GK has a prominent role as a glucose sensor because of its specific kinetic properties, including an affinity for glucose that is within the physiological plasma concentration range (half-saturation level for glucose [*S*_0.5_] of ~8 mmol/L), positive cooperativity, and lack of inhibition by glucose-6-phosphate. In liver and brain, GK is controlled by an endogenous inhibitor, the glucokinase regulatory protein (GKRP) [4, 5]. During starvation, the enzyme is bound to GKRP, leading to its inactivation and sequestration in the nucleus. After refeeding, glucose mediates the dissociation of the GK-GKRP complex and GK translocates to the cytoplasm. Also, several naturally occurring activating mutations have been described that induce a conformational change in GK, which results in higher affinity for glucose, and reduce the interaction between GK and GKRP [6, 7]. One such mutation, A456V, completely impairs GK-GKRP complex. Similarly, synthetic GK activators (GKAs) enhance GK activity and induce its translocation to the cytosol. [8–12].

There is evidence that a defect in GKRP regulation of GK, resulting in increased liver GK activity probably as a result of increased cytosolic translocation, has consequences on glucose and triglyceride homeostasis in humans, even though these patients have reduced risk to type 2 diabetes [13, 14].

We aimed to study the metabolic consequences of GK-GKRP deregulation by overexpressing a GK activating mutant (GK_A456V_) in the liver of insulin-deficient mice (lacking endogenous GK). Transfected GK_456V_ is maintained in the cytosol of hepatocytes * in vivo* leading to improved glycemia in the absence of dyslipidemia. These data provide novel insights into the consequences of GK-GKRP derangement and widen the scope for GKA research in the liver.

## 2. Materials and Methods

### 2.1. Directed Mutagenesis

The cDNA Encoding Rat GK Was Kindly Provided by Dr. Fatima Bosch (CBATEG, Universitat Autònoma de Barcelona). Site-Directed Mutagenesis to Generate GK-A456V cDNA Was Performed Using the QuickChange Site-Directed Mutagenesis Kit (Strategene), and Specific Primers Containing the Desired Mutation (3′CTGGTCTCTGCGGCGGCCTGCAAGAAG5′ and 5′GACCAGAGACGCCGCCGGTGCAAGAACG3′) from Dharmacon. Successful Incorporation of the Mutation Was Confirmed by DNA Sequence Analyses.

### 2.2. Plasmid Construction and Kinetic Analysis of Expressed GKs

The cDNAs for GK and GK-A456V were cloned into *Eco*RI-*Bgl*II of a pCAGGs vector which contains the CAG (cytomegalovirus immediate-early enhancer-chicken *β*-actin hybrid) promoter [15]. The resulting plasmids pGK and pGK_A456V_ were amplified in *E. coli* JM109 cells and extracted using NucleoBond PC 2000 EF Mega Prep kit (Macherey-Nagel). DNA for animal injection was digested with *SalI* and *HindIII* which cut on both sides of the expression cassette [16, 17] in order to extend expression *in vivo*. Later, DNA was purified by phenol/chloroform extraction. Expression plasmids were transfected in HuH7 cells for kinetic analysis (Supplementary Figure 1 available at doi: 10.1155/2011/707928).

### 2.3. Animal Care and Treatment

Male ICR mice purchased from Harlan Interfarma IBERICA S.L were maintained in a constant 12-hour light-dark cycle and fed standard rodent chow and water *ad libitum*. All animal protocols were approved by the Ethics Committee at the University of Barcelona. To obtain diabetic animals, mice weighing approximately 22 g were injected i.p. with a single dose of streptozotocin (STZ) (200 mg/kg) dissolved in 100 mM citric/citrate buffer, pH 4.5. One week later, glycemia was assessed after a 5-hour fast. Only those mice that had glycemia over 350 mg/dL were used. Digested plasmid DNA was introduced into murine livers using a hydrodynamic-based gene transfer technique via rapid injection of a large volume of DNA solution through the tail vein [18]. Briefly, 60 *μ*g/mouse DNA was diluted in 2.0 to 2.5 mL of apyrogenic Ringer-lactate solution (Fresenius Kabi) (0.1 mL/g body weight) and injected into the tail vein using a 27-gauge needle and syringe within 5 to 10 seconds. Typically, the mice recovered from the injection within 2 to 10 min. At the end of the experiment, animals were sacrificed under xylazine/ketamine anesthesia. Blood samples were taken by inferior cava vein puncture and serum was obtained by centrifugation at 700 ×g at 4°C for 15 min. Liver was dissected, snap frozen in liquid nitrogen, and subsequently stored at −80°C until analysis.

### 2.4. Immunohistochemistry

For GK immunodetection, livers from 5-hour fasted animals were fixed in a 4% PFA solution and frozen in OCT compound. 7 *μ*m cryosections were obtained in a cryostat and immunostained with a 1 : 50 dilution of GK antibody (AP7901c, Abgen) followed by an anti-rabbit antibody Alexa Fluor 488 (Molecular Probes).

### 2.5. Blood Metabolite Assays

Blood glucose levels were measured using a Glucocard Memory 2 apparatus (Menarini) from a blood drop collected from the tail tip. The Veterinarian Clinical Biochemistry Service from the Veterinary Hospital of the Universitat Autònoma de Barcelona, Bellaterra, Spain, measured serum metabolites. Serum insulin was determined using Ultrasensitive Mouse Insulin ELISA (Mercodia AB).

### 2.6. RNA Extraction and Quantitative

*RT-PCR *Total RNA was extracted with RNAeasy mini kit (Quiagen). cDNA synthesis was performed using Ready-To-Go You-Prime First Strand Beads (Amersham Biosciences) with random hexamers. mRNA levels of the transgenic rat GK (r-GK) were quantified with specie-specific primers (Applied Biosystems) and normalized using *β*-2-microglobulin as an internal control in an HT7700 Real Time-PCR system (Applied Biosystems). Data analysis was based on the ΔΔC_*t*_ method (Applied Biosystems).

mRNA levels of selected genes of interest were measured running a Low Density Array (Applied Biosystems) in an HT7900 Real Time-PCR system (Applied Biosystems). Expression was normalized using *β*-2-microglobulin as an endogenous control. Data analysis followed the ΔΔC_t_ method.

### 2.7. GK Activity

Frozen liver samples were homogenized in ice-cold homogenization buffer (HEPES 50 mM pH 7.4, KCl 100 mM, EDTA 1 mM, MgCl_2_ 5 mM, DTT 2.5 mM) using a polytron PT 3000 and centrifuged at 100,000 ×g for 1 hour at 4°C. Supernatants were used for the determination of GK activity following the spectrophotometric method described by Davidson and Arion [19]. Briefly, the assays were carried out in a solution containing HEPES 50 mM pH 7.4, KCl 100 mM, MgCl_2_ 7.5 mM, NAD^+^ 1 mM, ATP 5 mM, BSA 1% and indicated amounts of glucose. The reactions were initiated by adding 5 U/mL of glucose-6-phosphate dehydrogenase (G6PDH) from *Leuconostoc mesenteroides* (Roche) and the rate of increase in absorbance at 340 nm was measured. The GK activity was calculated as the difference between the kinase activities measured at different glucose concentrations and 0.5 mM glucose (hexokinase activity), and was expressed as mUnits/mg liver.

### 2.8. G6PDH Activity

Frozen liver samples were homogenized in ice-cold buffer, which contained HEPES 50 mM pH 7.4, KCl 100 mM, EDTA 1 mM, MgCl_2_ 5 mM, DTT 2.5 mM, and 1% pluronic F-127 (Calbiochem), and centrifuged at 20,000 ×g for 20 min at 4°C. G6PDH activity was assayed by measuring the rate of NADPH production from NADP^+^ 0.5 mM and glucose-6-phosphate 2 mM, at pH 7.6 [20]. Results were expressed as mUnits/mg protein in the supernatant. Protein was measured using the Bradford method (Bio-Rad).

### 2.9. Western Blot

Frozen tissue was homogenized in radioimmunoprecipitation assay buffer (RIPA) supplemented with protease and phosphatase inhibitors and centrifuged at 15,000 ×g for 15 min at 4°C. 30 *μ*g of tissue extract was resolved by SDS-PAGE (8–12% gel) and electrotransferred onto a Hybond-P membrane (Amersham Biosciences). Antibody against GK (H-88, Sta Cruz) was used at 1/500 dilution. 1/1000 dilution was used for the following antibodies: 6-phosphofructo-2-kinase/fructose-2,6-biphosphatase (ub-PFK2) and L-pyruvate kinase (L-PK) (gifts from Ramon Bartrons, Barcelona University), and Carbohydrate Response Element Binding Protein (CHREBP) (Novus Biologicals). ACC and ACC-P Ser-79 (Upstate) were used at 1/2000. Sheep anti-PEPCK-C antiserum (a gift from Daryl Granner, Vandervilt University, USA) was used at 1 : 20,000 dilution. All membranes were normalized using monoclonal anti-*γ*-tubulin (Sigma) at 1 : 10,000 dilution. Horseradish peroxidase activity linked to secondary antibody was detected with ECL substrate (Pierce) in a Fujifilm LAS 300 Intelligent Dark Box IV imaging system. Densitometry was performed using Multi Gauge Software.

### 2.10. Hepatic Metabolites

To measure hepatic free glucose, glucose-6-phosphate (G6P), and lactate, acid homogenates were obtained from frozen liver. Briefly, perchloric acid 10% was added to frozen pieces of liver (5 : 1 v : w) and the mixture was homogenized in a Potter Elvehjem apparatus at 1,500 rpm. The homogenate was then centrifuged at 20,000 ×g for 10 min at 4°C. The supernatant was brought to pH 7 with K_2_CO_3_ 4 N and centrifuged again to remove the KClO_4_ formed during neutralization. Free glucose was measured using a Glucose Oxidase kit (Sigma). G6P was measured following the spectrofluorometric method described by Lang & Michal, which is based in the reaction catalyzed by G6PDH. Briefly, the assay solution contained triethanolamine 0.2 M pH 7.6, NADP^+^ 0.2 M, MgCl_2_ 5 mM, and G6PDH 0.17 U/mL. Lactate was determined by a spectrophotometric method based on the reaction catalyzed by lactate dehydrogenase, following Gutmann & Wahlefeld. Briefly, the reaction cuvette contained hydrazine/glycine buffer 0.25 M/0.7 M, pH 9.3, EDTA 0.15 mM, NAD^+^ 2 mM, and 35 U/mL of lactate dehydrogenase.

For fructose-2,6-bisphosphatase (F2,6bP) determination, frozen livers were homogenized in 0.1 M NaOH, heated to 80°C for 15 min, and centrifuged at 12,000 ×g for 5 min. F2,6bP was determined in supernatants by its ability to activate pyrophosphate-dependent 6-phosphofructo-1-kinase from potato tubers, as described by Van Schaftingen et al. [21].

The hepatic triacylglyceride (TAG) content was measured in 3 M KOH, 65% ethanol extracts using a TAG kit (Sigma), based on the method of Salmon and Flatt for liver saponification.

Hepatic glycogen content was measured essentially as described elsewhere [22].

### 2.11. Whole-Body Glucose Utilization

A solution of U-^14^C-glucose and glucose was intraperitoneally injected into 5-hour fasted STZ-diabetic mice (0.2 mg glucose, 10 *μ*Ci per animal). Blood, liver, brain, and muscle were obtained 30 minutes after injection and dissolved into Solvable (Perkin Elmer). Blood samples were bleached with EDTA 0.1 M and H_2_O_2_ (30%), and liver and brain samples were bleached with H_2_O_2_ (30%) alone. Radioactivity was quantified by adding scintillation liquid (Ecoscint H) and counted in a Wallac 1409-001 counter.

### 2.12. Statistical Analysis

Results are expressed as the mean ± standard error (SE). Statistical analysis was performed by one-way ANOVA with Bonferroni posttest. Differences were considered significant when *P* < 0.05. 

## 3. Results

### 3.1. Liver Overxpression of pGK and pGK_A456V_

pGK_A456V_, wild-type GK (pGK) and an empty vector (pControl), were introduced into the liver of streptozotocin (STZ-) treated mice. Hydrodynamic-based delivery of plasmid DNA led to specific transfection of hepatocytes around the central vein [23, 24], reproducing the physiologic distribution of GK in the liver acinus [25]. This technique induces a transient release of liver enzymes that returns to control values by the second day after injection. Consistently, transaminase levels were low at the end-point of analysis, and similar in all treatment groups (pControl 207.5 ± 29.88 U/L, pGK 121.0 ± 48.43 U/L, pGK_A456V_ 117.5 ± 40.35 U/L; *n* = 4–9).

**Figure 1:**
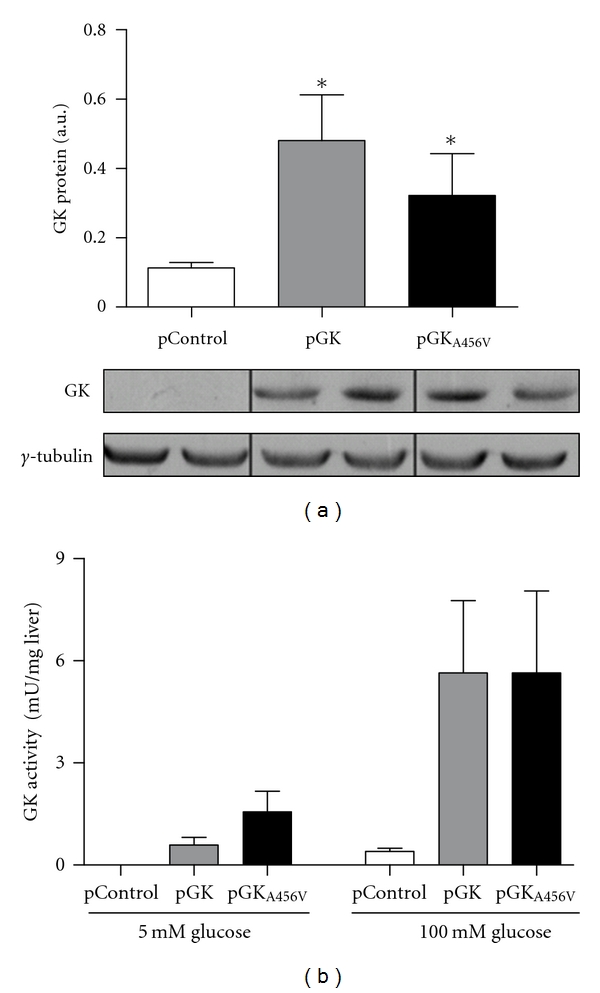
Rat GK and GK_A456V_ overexpression in the livers of STZ-treated mice. 60 *μ*g of pControl, pGK, or pGKA_456V_ were hydrodynamically-transfected to diabetic mice. (a) GK protein content of liver homogenates was determined by Western blot. A representative blot and densitometric analysis is shown. (b) Further confirmation of GK overexpression was obtained by analysis of specific GK activity in liver extracts at different glucose concentration. Values are expressed as the means ± SE (*N* = 4. *P < 0.05 versus pControl).

At the time of injection mice were hyperglycemic (>350 mg/dL) and their insulin level was undetectable (<0.1 *μ*g/L). Forty-eight hours postinjection the expression of transfected rat GK and GK_A456V_ was detected by RT-PCR (data not shown). Further confirmation of GK overexpression was obtained by Western blot from liver extracts (Figure 1(a)) and GK activity assay (Figure 1(b)). GK activity was undetectable in pControl transfected livers irrespective of glucose concentration, as expected in long-term STZ diabetic animals. In contrast, both pGK and pGK_A456V_ transfected groups had similar GK activity at 100 mM glucose (mU/g fresh), demonstrating that both treatment groups had similar amounts of GK protein. Consistent with the *S*_0.5_ for glucose of GK_A456V_, liver extracts from pGK_A456V_-injected animals had twice the GK activity of livers from pGK-injected animals at physiological glucose concentrations (5 mM) (Figure 1(b)).

### 3.2. Different Compartmentalization of GK and GK_A456V_

**Figure 2:**
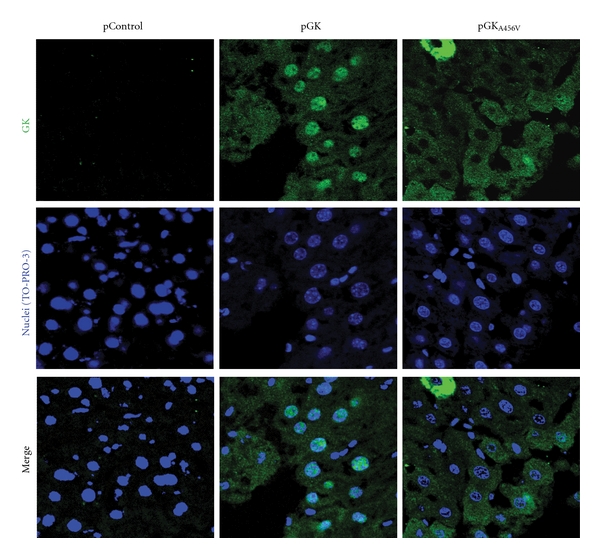
GK and GK_A456V_ localization inside hepatocytes. Liver sections of 5-hour fasted mice injected with pControl, pGK, or pGK_A456V_ were immunostained with GK antibody. TO-PRO-3 was used to visualize nuclei.

GKRP retains GK in the nucleus at low glucose concentrations and releases GK when insulin, high glucose, or fructose-1P are present [26, 27]. Different studies have suggested that the A456V mutation impairs GKRP regulation *in vitro *[6, 28]. Therefore, we assessed GKRP-dependent nuclear sequestration in primary hepatocytes from STZ-treated rats transfected with pGK and pGK_A456V_, in the presence or absence of pGKRP. Densitometry analysis of Western blots from nuclear and cytosolic fractions showed that cellular GK_A456V_ distribution was not affected by the presence of GKRP (data not shown). We next examined the compartmentalization of GK and GK_A456V_ *in vivo* by immunohistochemistry. pGK-treated mice showed GK immunoreactivity throughout the liver, especially in nuclei (Figure 2). Interestingly, overexpressed GK_A456V_ protein was localized predominantly in the cytosol of transfected hepatocytes. These data demonstrates, that impaired GKRP binding to GK_A456V_ changes its compartmentalization *in vivo*.

### 3.3. Glucose Homeostasis in Fed Animals

**Figure 3:**
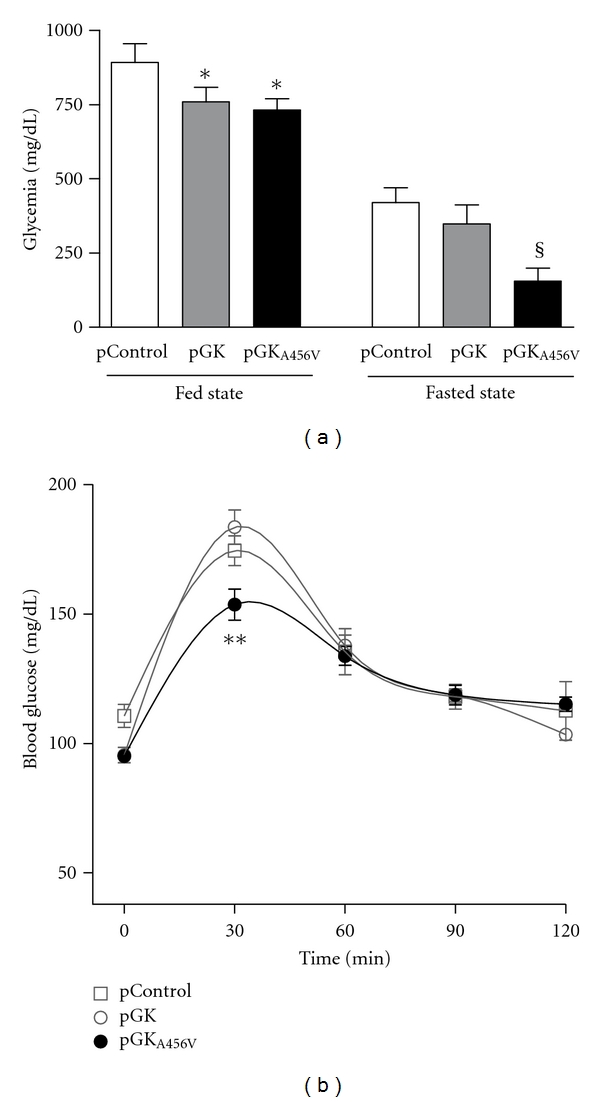
Blood glucose homeostasis in GK- and GK_A456V_-treated mice. Streptozotocin-induced diabetic mice were hydrodynamically injected with of pControl, pGK or pGK_A456V_. (a) Blood glucose levels were determined 48 h posttransfection in fed state and after a 5-hour fast. Values are the mean ± SE (*N* = 5–12, *P < 0.05 versus pControl, ^§^ *P* < 0.05 versus pControl and versus pGK). (b) Glucose tolerance test in overnight fasted, healthy mice transfected with pControl, pGK, or pGK_A456V_. **AUC versus pControl and pGK.

**Figure 4:**
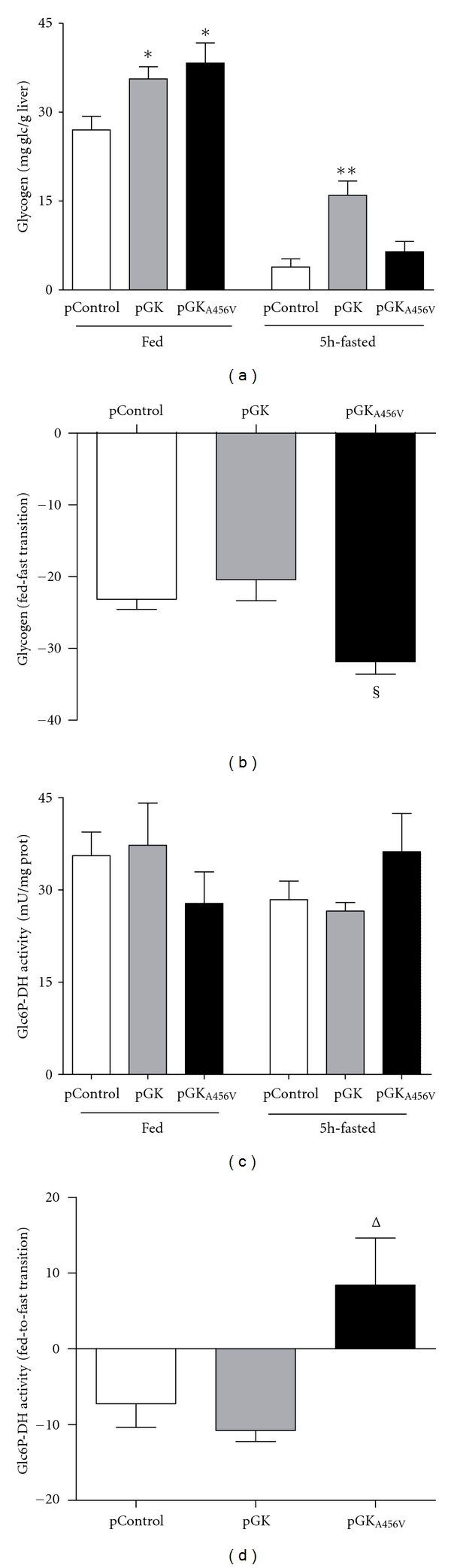
Effects of GK and GK_A456V_ overexpression on glycogen and glucose-6-phosphate DH activity in the liver. (a) Glycogen levels from fed and 5-hour fasted mice. (b) Glycogen balance between the fast and fed transition. (c) Glc6P DH activity was measured in liver extracts of mice in fed and fasted state. (d) Variation of Glc6P DH activity during the fed-to-fast transition. Data are mean ± SE. (*N* = 5–9. *P < 0.05 versus pControl; ^§^ *P* < 0.05 versus pControl and *P* < 0.01 versus pGK; Δ*P* < 0.05 versus pControl and pGK).

The impact of both GK enzymes on glucose metabolism was first evaluated in fed animals. Both GK and GK_A456V_ slightly reduced glycemia (Figure 3(a)). To explore the fate of glucose in the liver, we measured several metabolites in hepatic extracts (Table 1). Fed pGK-treated mice showed significantly higher F2,6BP and glycogen contents (Figure 4(a)), together with a rise in lactate, indicating a higher glycolytic flux. GK_A456V_-expressing livers also showed higher hepatic free glucose than both control and GK groups, even though the content of G6P was similar in all three groups. Furthermore, both F2,6BP and lactate content were increased. Glycogen content was significantly higher than control but similar to pGK-treated group (Figure 4(a)).

### 3.4. Glucose Homeostasis in 5-Hour Fasted Animals

pGK_A456V_-treated animals showed a marked reduction of glycemia after a 5-hour fast (Figure 3(a)). Lactate was only increased after pGK treatment (pControl, 7.296 ± 0.772 mM; pGK_A456V_, 8.778 ± 0.649 mM; pGK, 10.62 ± 0.608 mM—*P* < 0.05 versus pControl and pGK_A456V_—). Hepatic free glucose content was low in the pGK_A456V_ treatment group (Table 1). G6P levels were reduced to the same extent in control and GK_A456V_ groups, whereas GK-expressing animals showed similar G6P and ATP concentrations to well-fed animals. F2,6BP and lactate levels remained higher in both treatment groups as compared to control. Glycogen content in GK-expressing animals was significantly higher than control and GK_A456V_-expressing groups. Fed-to-fast transition produced a marked reduction of glycogen stores in pGK_A456V_-treated animals as compared to pControl and pGK, suggesting an increased rate of glycogenolysis in GK_A456V_-expressing animals (Figure 4(b)). In order to clarify whether glucose distribution *in vivo* favored the liver, we quantitated ^14^C radioactivity derived from uptake and metabolism of U^14^C-glucose in various tissues 30 minutes after injection. ^14^C label was increased exclusively in the liver of both pGK and pGK_A456V_ treatment groups (control, 100 ± 7.88%; GK, 138.73 ± 14.63%; GK_A456V_ 144.1 ± 8.99%; *P* = 0.037 GK versus control and *P* = 0.003 pGK_A456V_ versus control). To assess whether pGK_A456V_ expression would affect glucose clearance in healthy mice, we performed a glucose tolerance test 1 week after transfection (Figure 3(b)).

Liver GK overexpression has been associated with dislipidemia [29]. Consistently, our data demonstrates a significant increase in serum TAG (pControl 33.5 ± 7.72 mg/dL, pGK_A456V_ 38.13 ± 7.66 mg/dL, pGK 74.00 ± 14.76 mg/dL, *P* < 0.05 versus pControl and pGK_A456V_) and NEFA (pControl 0.325 ± 0.09 mM, pGK_A456V_ 0.4013 ± 0.05 mM, pGK 0.66 ± 0.10 mM *P* < 0.05 versus pControl) only in the postabsorptive state. Besides, GK overexpressing mice showed a tendency to increase hepatic TAG content, which is consistent with the literature [30, 31]. In contrast, GK_A456V_ overexpression did not promote dislipemia, as evidenced by maintained levels of circulating NEFA and TAG. There was a significant increase in *β*-hydroxybutyrate levels in the pGK_A456V_ group (pControl, 0.18 ± 0.03 mM; pGK_A456V,_ 0.27 ± 0.03 mM—*P* < 0.05 versus pControl; pGK, 0.20 ± 0.01 mM).

**Figure 5:**
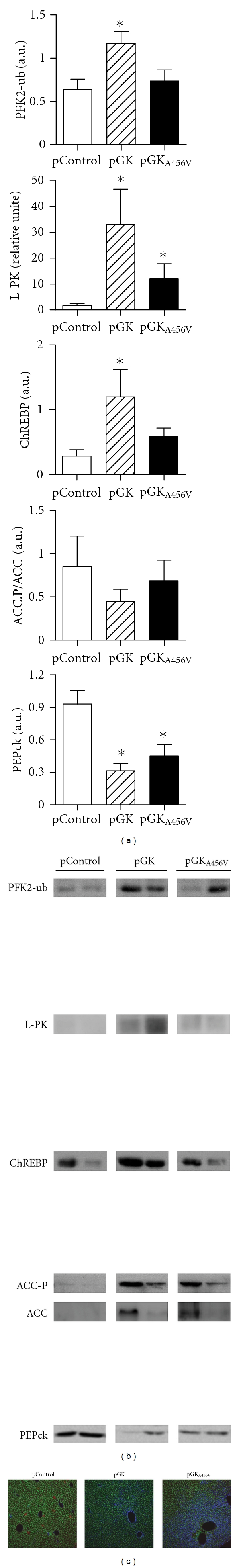
Analysis of protein levels in livers transfected with GK and GK_A456V_. Livers excised from mice in postabsorptive state were homogenized and resolved by Western blot. (a, b) Representative blots from three independent experiments. The densitometric analysis is presented (*N* = 4, *P < 0.05 versus pControl, ***P < 0.001 versus pGK_A456V_). (c) Liver sections of 5-hour fasted mice injected with pControl, pGK, or pGK_A456V_ were immunostained with Glc6Pase antibody. TO-PRO-3 was used to visualize nuclei.

In order to elucidate the mechanism responsible for the metabolic profile observed, we analyzed protein and mRNA levels for key enzymes and transcription factors. Both GK and GK_A456V_ treatments were accompanied by increases in L-PK protein (Figures 5(a) and 5(b)). pGK-treatment resulted in higher levels of ubiquitous PFK2 (*Pfkfb3*) protein (Figures 5(a) and 5(b)), but not mRNA (Table 2), consistent with elevated F2,6BP observed in this treatment group (Table 1). Also, endogenous, mouse GK mRNA expression was induced in the livers of pGK-injected mice, as detected by species-specific qRT-PCR. Correlating with the induction of the expression of endogenous GK, cMyc mRNA was only increased in pGK-treated animals. Other regulatory factors implicated in the regulation of endogenous GK expression, such as SREBP and LXR*α*, were not affected by treatment with pGK and pGK_A456V_ (Table 2).

As an increment in GK expression in the liver is commonly associated with increased lipogenesis [32, 33], enzymes and transcription factors involved in *de novo *lipogenesis were analyzed. GK- and pGK_A456V_-expressing groups presented increased mRNA content for Fasn and Mod1 that were more pronounced in pGK-treated animals. Also, Acc1 and Chrebp were more induced by the overexpression of GK than by GK_A456V_.

GK, through the production of xylulose-5P, mediates the inhibition of gluconeogenesis by glucose [34, 35]. F2,6BP [36] has also been associated with the inhibitory effect of glucose metabolism on gluconeogenesis. Consistently, we observed a reduction of PEPCK at the protein level in both pGK- and pGK_A456V_-treated mice. However, PEPCK mRNA was reduced only in GK-overexpressing animals. Similarly, PGC-1*α* and HNF4*α* mRNAs were reduced in pGK-injected mice, whereas GK_A456V_-overexpressing livers only showed reduced PGC-1*α* mRNA.

In contrast, the level of mRNA for the catalytic subunit of glucose-6-phosphatase (Glc6Pase) were strikingly higher in GK_A456V_-overexpressing animals, consistent with more glycogen breakdown observed after a 5-hour fast. Glc6Pase protein was also increased as assessed by immunohistochemistry (Figure 5(c)). Similar results were observed in healthy mice overexpressing pGK_A456V_ after a short fast (data not shown).

## 4. Discussion

To evaluate the consequences of GK activation and deregulation by GKRP in the liver, we hydrodynamically injected pGK_A456V_ in the liver of STZ-diabetic mice. This strategy allowed us to obtain a mouse model where the effects of exogenous GKs are isolated from those of endogenous GK (undetectable levels of mouse GK mRNA and activity in pControl-treated animals) and insulin action (insulin levels <0.1 *μ*g/L in all treatment groups). In addition, the use of hydrodynamic gene transfer provides proper zonal expression of GK and GK_A456V_ in perivenous hepatocytes [23, 25]. The efficiency of this gene transfer technique is variable. Therefore, only animals that were positive for exogenous expression of GK as determined by qRT-PCR (3x SD of controls) were selected for further analysis (data not shown). After selection, both GK-overexpressing groups had identical protein levels and maximal activity (100 mM glucose) Figures 1(a)–1(b). These results are not in agreement with the reported instability of liver GK protein in a PHHI-GK_A456V_ transgenic mouse [28] and the GKRP knock-out model [37, 38], probably because of the lack of endogenous transcriptional control in pGK_A456V_. Also, GK_A456V_ activity at 5 mM glucose was higher in liver extracts, as expected from the kinetic changes associated with the mutation.

The GK_A456V_ mutation resides on a hinge of the enzyme responsible for the conformational changes associated with GKRP interaction and allosteric activation. GKRP regulates GK action in hepatocytes by retaining the enzyme in the nucleus at low glucose concentrations and liberating GK when insulin, high glucose levels, or fructose-1P is present [26, 27]. We show here that GK_A456V_ is predominantly cytosolic in the liver of transfected animals, contrary to the expected nuclear compartmentalization of wild-type GK. In addition, GK_A456V_ was not appropriately translocated to the nucleus of diabetic primary hepatocytes in the presence of excess GKRP (data not shown). Lack of GKRP binding to GK_A456V_ has been shown by Heredia et al. [6] *in vitro* and inferred from the structural characterization of GK bound to synthetic activators [12, 39]. Recent reports on the P446L mutation in GKRP also suggest that a decrease in GK inhibition results in increased GK activity, suggesting that GK localizes to the cytosol [13].

An analysis of GK and GK_A456V_ overexpression in well-fed animals demonstrated small but significant changes in glycemia, together with increased hepatic glucose metabolism, as suggested by higher F2,6BP and glycogen content, in the absence of alterations to lipid serum metabolites. No changes were observed in enzymes or regulatory proteins involved in gluconeogenesis in fed animals (data not shown). These results are partially consistent with the phenotype observed by Morral et al. [31, 33, 40], who used a low dose of GK-expressing adenovirus (expressed largely in periportal hepatocytes, [41, 42]) but differ from those obtained using high doses of adenovirus, indicating that dose and/or zonal expression of GK influences the metabolic impact of GK overexpression.

When animals were examined after a short fast (postabsorptive), marked differences were observed among groups.

(1) GK overexpression reduced glycemia ~15%, consistent with enhanced glucose metabolism in the liver, as shown by increased G6P, F2,6BP, glycogen content, and glycolytic and lipogenic enzymes together with higher serum lactate, NEFA and TAG concentrations. Matsuzaka et al. [43] have shown that a fed-to-fast transition in STZ-diabetic mice results in increased gluconeogenic potential and fatty acid oxidation in the liver, consistent with results obtained in pControl-treated animals in the present study. Conversely, pGK treatment resulted in reduced PEPCK and PGC1*α* mRNA, supporting the view that GK overexpression indirectly inhibits gluconeogenesis and fatty-acid oxidation. Consistently, we measured higher NEFA and TAG serum levels in the postabsorptive state although no changes in *β*-hydroxybutyrate were observed. These results indicate that enhanced glucose metabolism resulting from GK overexpression had a small effect on glycemia, possibly because pGK dose was low.

(2) GK_A456V_ overexpression led to a significant reduction of serum glucose of ~60% that was consistent with higher glucose clearance on a glucose tolerance test in fasting, healthy mice. Fasting hypoglycemia is also observed in human carriers of the P446L GKRP mutation [14, 44]. GK_A456V_ overexpression did not impact the concentration of serum lipids whereas *β*-hydroxybutyrate was increased, contrary to the dyslipidemia observed in GK overexpressing animals in this and earlier studies, including human carriers of the P446L GKRP mutation [14, 44]. Both kinetic and regulatory issues might account for the observed differences.

Hepatic levels of F2,6BP and L-PK protein were increased but other indicators (G6P, Fasn mRNA, ChREBP protein, and mRNA and glycogen content) of glucose utilization were lower than in GK-expressing livers. Importantly, glycogen balance (fed-to-fast) was significantly lower in GK_A456V_-expressing animals, indicating an important net loss of glycogen. G6Pase expression was also higher in this group of animals, providing an explanation for increased glycogen loss observed in GK_A456V_-containing livers. The involvement of G6Pase in the positive regulation of glycogenolysis has been shown in several animal models overexpressing P36 or P46 components of the G6Pase system, resulting in decreased glycogen levels in the hepatocyte [45–48]. Moreover, a lack of G6Pase system activity observed in glycogen storage disease type 1a, or in a G6Pase KO promotes glycogen accumulation [49]. Activation of the nonoxidative branch of the pentose phosphate pathway, as a result of high exogenous glucose, transiently upmodulates G6Pase mRNA levels and stability in an insulin-independent manner [34, 35]. GK_A456V_-expressing livers showed higher G6PDH activity and GSH/GSSG ratio during the fed-to-fast transition than control or GK groups (Figures 4(c)-4(d) and Table 1). Increased G6Pase mRNA and protein in the presence of GK_A456V_ would suggest an induction of glucose cycling. The physiological explanations for this futile pathway are not completely understood. It is, however, clear that this counter regulation shall have consequential effects on glucose homeostasis if persistent, as occurs in the GK_A456V_ model.

All in all, the present data confirms that pGK overexpression in the liver results in relatively small changes in glycemia but overt dislypidemia. The present model represents the first attempt to overexpress pGK in perivenous hepatocytes, and confirms previous observations using adenovirus [29]. In contrast to the work of Scott et al. [50] and Morral et al. [40], GK overexpression in perivenous hepatocytes does not significantly affect Glc6Pase expression, suggesting that zonation is an important experimental variable not sufficiently addressed to date in the field. In addition, we have demonstrated that GK_A456V_ overexpression reduces blood glucose to a great extent, probably due to its capacity to induce Glc6Pase.

We conclude that impaired GKRP regulation and GK_A456V_ altered kinetics greatly influence liver metabolism, in line with results obtained in humans with a mutant GKRP. Besides, it suggests that activating GK exclusively in the liver could be a feasible strategy for diabetes, widening the scope for GKA research. Similar conclusions, based on its potent induction of glucose clearance by the liver, have been obtained after liver-specific GK activation in chicken in a recent study by Rideau et al. [51]. Studies on animal models of type 2 diabetes are under way in our laboratory in order to evaluate the metabolic impact in the context of insulin resistance and obesity.

## Supplementary Material

Supplementary figure 1. pGK and pGKA_456V_ analysis in vitro. (A) Comparision between glucokinase activity at different glucose concentrations of hepatoma cells Huh-7 which have been transfected with pGK (continuous line) and pGKA_456V_ (discontinuous line), respectively. (B) Activity homogenates were also used to evaluate through Western Blot the expression levels of pGK and pGKA_456V_. “pC” lane corresponds to an homogenate of Huh-7 cells transfected with a pEGFP plasmid and “+” is the positive control and consists of a fed mouse liver homogenate.

## Figures and Tables

**Table 1:** Liver metabolites of STZ-diabetic mice transfected with pControl, pGK and pGK_A456V_.

	Fed			5 h-Fasted		
	pControl	pGK	pGK_A456V_	pControl	pGK	pGK_A456V_
Glc (*μ*mol/g liver)	40.70 ± 5.921	32.72 ± 1.475	47.68 ± 7.328	52.29 ± 9.524	32.23 ± 12.18	6.35 ± 4.043*
	*n* = 11	*n* = 6	*n* = 9	*n* = 5	*n* = 4	*n* = 4
G6P (*μ*mol/g liver)	0.16 ± 0.021	0.14 ± 0.018	0.15 ± 0.021	0.06 ± 0.024	0.20 ± 0.068^§^	0.04 ± 0.017
	*n* = 11	*n* = 8	*n* = 10	*n* = 5	*n* = 4	*n* = 5
F2,6BP (nmol/g liver)	1.19 ± 0.215	2.84 ± 0.625*	1.90 ± 0.517	0.71 ± 0.173	9.86 ± 1.812^§^	4.14 ± 1.607
	*n* = 11	*n* = 8	*n* = 10	*n* = 5	*n* = 5	*n* = 5
Lactate (*μ*mol/g liver)	1.31 ± 0.275	1.78 ± 0.424	2.27 ± 0.951	1.16 ± 0.239	1.28 ± 0.322	1.32 ± 0.222
	*n* = 12	*n* = 8	*n* = 10	*n* = 5	*n* = 4	*n* = 5
TAG (mg/g liver)	6.25 ± 0.26	6.08 ± 0.36	6.26 ± 0.17	9.18 ± 1.48	12.56 ± 0.50	11.84 ± 2.13
	*n* = 12	*n* = 8	*n* = 10	*n* = 5	*n* = 5	*n* = 5
GSH/GSSG ratio	11.73 ± 0.87	11.81 ± 0.93	11.37 ± 0.87	5.372 ± 0.49	9.479 ± 1.38	9.926 ± 1.84*
	*n* = 12	*n* = 8	*n* = 10	*n* = 5	*n* = 5	*n* = 5

Data are means ± SE. (*N* = 4–12). *P < 0.05 versus its pControl, ^§^ *P* < 0.05 versus fast pControl and fast pGK_A456V_.

**Table 2:** Gene expression profile in livers of diabetic mice after a short fast.

Gene	Protein	pControl	pGK	pGK_A456V_
Glycolysis and lipogenesis				
Gck	GK	1.26 ± 0.72	5.28 ± 1.49	1.73 ± 0.86
Pfkfb3	PFK2	1.05 ± 0.13	1.17 ± 0.19	1.22 ± 0.19
Nr1h3	Lxr-*α*	1.03 ± 0.11	0.81 ± 0.03	0.91 ± 0.05
Fasn	FAS	1.14 ± 0.21	11.37 ± 5.76*	2.33 ± 0.42
Mod1	ME	1.05 ± 0.13	2.43 ± 0.91	1.57 ± 0.32
Myc	c-myc	1.07 ± 0.26	3.29 ± 1.49	0.94 ± 0.19
Srebf1	SREBP1c	1.03 ± 0.10	0.90 ± 0.16	0.85 ± 0.11
Chrebp	CHREBP	1.08 ± 0.12	2.31 ± 0.72	1.90 ± 0.29

Gluconeogenesis				
Pck1	PEPCK-C	1.04 ± 0.12	0.52 ± 0.11*	0.91 ± 0.16
G6pc	Glc-6Pase	0.95 ± 0.31	1.70 ± 0.56	4.52 ± 1.39^§^
Hnf4	HNF-4*α*	1.05 ± 0.13	0.68 ± 0.07	0.77 ± 0.12
Slc2a2	GLUT-2	1.01 ± 0.05	1.20 ± 0.25	1.01 ± 0.12

Energy metabolism				
Cpt1a	Cpt-1*α*	1.02 ± 0.09	0.83 ± 0.08*	0.92 ± 0.08
Hmgcs2	HmgCoA Synthase	1.02 ± 0.08	1.14 ± 0.22	0.91 ± 0.11
Mdh1	cMDH	1.01 ± 0.05	0.74 ± 0.04*	0.70 ± 0.10 *
Ucp2	UCP-2	1.03 ± 0.10	0.66 ± 0.02*	0.96 ± 0.15
Ppargc1	PGC-1*α*	1.02 ± 0.07	0.27 ± 0.07^†^	0.63 ± 0.14 *

Each value on the table represents the mean relative amount of mRNA with respect to that in the control experiment treatment (mean ± SE). Liver gene expression profile was analyzed by qRT-PCR using Applied Biosystems 7900HT Micro Fluidic Card and ΔΔCt calculations. (*N* = 5 per group). *P < 0.05 versus pControl; ^§^ *P* < 0.05 versus pControl and pGK; ^†^ *P* < 0.001 versus pControl and *P* < 0.05 versus pGK_A456V_.
