# Supplementary figures and images for: Liver GlucokinaseA456V Induces Potent Hypoglycemia without Dyslipidemia through a Paradoxical Induction of the Catalytic Subunit of Glucose-6-Phosphatase
Source: Int J Endocrinol. 2011 Dec 13;2011:707928. doi: 10.1155/2011/707928 (PMC3238378; doi:10.1155/2011/707928)

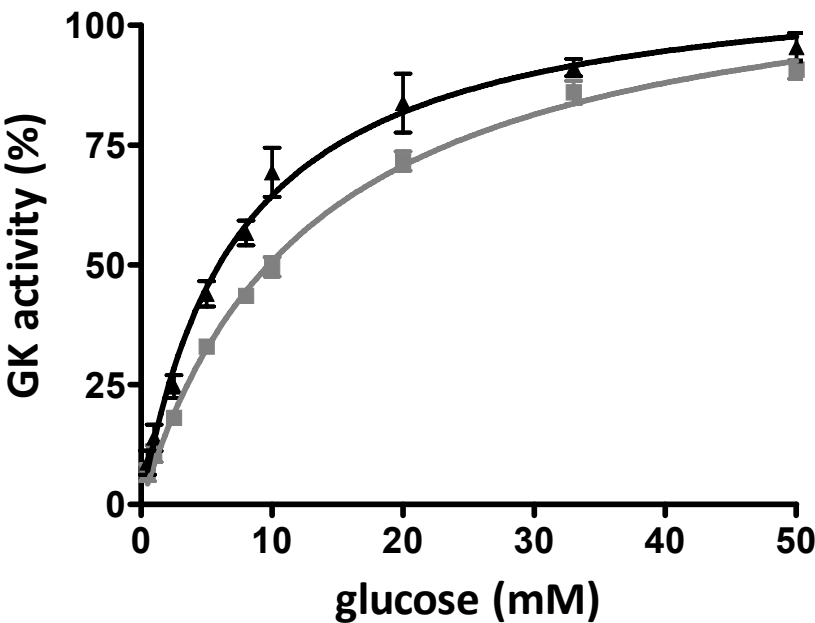

Supplement: Supplementary file 1 — Supplementary figure 1. pGK and pGKA456V analysis in vitro. (A) Comparision between glucokinase activity at different glucose concentrations of hepatoma cells Huh-7 which have been transfected with pGK (continuous line) and pGKA456V (discontinuous line), respectively. (B) Activity homogenates were also used to evaluate through Western Blot the expression levels of pGK and pGKA456V. “pC” lane corresponds to an homogenate of Huh-7 cells transfected with a pEGFP plasmid and “+” is the positive control and consists of a fed mouse liver homogenate. [file 707928.f1.pdf]
